# Antibiotic control of antibiotic resistance in hospitals: a simulation study

**DOI:** 10.1186/1471-2334-10-254

**Published:** 2010-08-25

**Authors:** Michael Haber, Bruce R Levin, Piotr Kramarz

**Affiliations:** 1 Department of Biostatistics and Bioinformatics, Emory University School of Public Health, Atlanta, GA, USA; 2 Department of Biology, Emory University, Atlanta, GA, USA; 3 Pfizer Ltd, Walton Oaks, U.K; 4 Current Address: the European Centre for Disease Prevention and Control (ECDC), Stockholm, Sweden

## Abstract

**Background:**

Using mathematical deterministic models of the epidemiology of hospital-acquired infections and antibiotic resistance, it has been shown that the rates of hospital-acquired bacterial infection and frequency of antibiotic infections can be reduced by (i) restricting the admission of patients colonized with resistant bacteria, (ii) increasing the rate of turnover of patients, (iii) reducing transmission by infection control measures, and (iv) the use of second-line drugs for which there is no resistance. In an effort to explore the generality and robustness of the predictions of these deterministic models to the real world of hospitals, where there is variation in all of the factors contributing to the incidence of infection, we developed and used a stochastic model of the epidemiology of hospital-acquired infections and resistance. In our analysis of the properties of this model we give particular consideration different regimes of using second-line drugs in this process.

**Methods:**

We developed a simple model that describes the transmission of drug-sensitive and drug-resistant bacteria in a small hospital. Colonized patients may be treated with a standard drug, for which there is some resistance, and with a second-line drug, for which there is no resistance. We then ran deterministic and stochastic simulation programs, based on this model, to predict the effectiveness of various treatment strategies.

**Results:**

The results of the analysis using our stochastic model support the predictions of the deterministic models; not only will the implementation of any of the above listed measures substantially reduce the incidences of hospital-acquired infections and the frequency of resistance, the effects of their implementation should be seen in months rather than the years or decades anticipated to control resistance in open communities. How effectively and how rapidly the application of second-line drugs will contribute to the decline in the frequency of resistance to the first-line drugs depends on how these drugs are administered. The earlier the switch to second-line drugs, the more effective this protocol will be. Switching to second-line drugs at random is more effective than switching after a defined period or only after there is direct evidence that the patient is colonized with bacteria resistant to the first antibiotic.

**Conclusions:**

The incidence of hospital-acquired bacterial infections and frequencies of antibiotic resistant bacteria can be markedly and rapidly reduced by different readily implemented procedures. The efficacy using second line drugs to achieve these ends depends on the protocol used for their administration.

## Background

Over the past two decades, antibiotic resistance has become an increasingly grave health problem, serious enough for some to see this not-unanticipated product of evolution as foretelling of the end of the antibiotic era [1]. Because of resistance, bacterial infections that had been readily cleared by antibiotics are lasting longer and are more likely to result in severe morbidity and mortality than they would be if these infecting bacteria were susceptible to the treating antibiotic(s). This resistance problem is particularly serious in hospitals, where patients are commonly compromised by age, illness and treatment with immune suppressing drugs. Invasive procedures and the use of life-support machinery that are likely to be infected by bacteria also contribute to antibiotic-resistant hospital infections [2]. Moreover, patients who enter hospitals for the treatment of resistant bacterial infections or acquire resistant infections while in the hospital are adding to the already too high costs of healthcare [3] and are a source of resistant bacteria and/or resistance-encoding genes.

Within this pessimistic framework, however, there is an element of optimism. Hospitals are potentially containable institutions in which the use of antibiotics can be monitored and managed. Unlike the open communities it should be, and in some countries like the Netherlands [4] has been, possible to control the spread of resistance in hospitals. In accord with mathematical models [5], the frequency of colonization and infections with antibiotic-resistant bacteria in hospitals can be reduced in at least five ways by: (1) reducing the rate of use of drugs for which there is resistance, (2) improving infection control and thereby reducing transmission between patients and from hospital care workers to patients, (3) increasing the rate of turnover of patients, (4) reducing the rate of influx of patients with resistant bacteria into hospitals or their intensive care wards, and (5) using additional antibiotics for which there is no resistance [6]. This theory predicts that not only will these measures individually and collectively reduce the frequency of resistant bacteria in hospitals, they will also reduce the absolute rate of these infections. Moreover, and most importantly, the effects of their implementation should be manifest over a relatively short period of time. Declines in the rates of infection and frequency of resistant bacteria should be seen in months rather than the years or decades anticipated for controlling resistance in open communities.

In this report, we consider the contribution of the different factors controlling the incidence of hospital-acquired infections and the spread of antibiotic resistant bacteria considered by [5], giving primary consideration to the use of second-line antibiotics for which there is no resistance. Using a deterministic and a stochastic simulation model, we explore the effects of different regimes employing these second-line antibiotics to successfully treat patients and, at the same time, reduce the frequency of resistance to other drugs. Treatment is initiated with the first-line antibiotic, for which there is resistant bacteria. Patients may be switched to the second-line drug in one of three ways: (i) at random, with a constant probability of switching each day, (ii) after a defined term with the first-line drug, and (iii) directed, where the patient remains on the first-line drug until testing provides evidence that she/he is colonized with bacteria resistant to that drug.

## Methods

### The Basic Model

**Figure 1:**
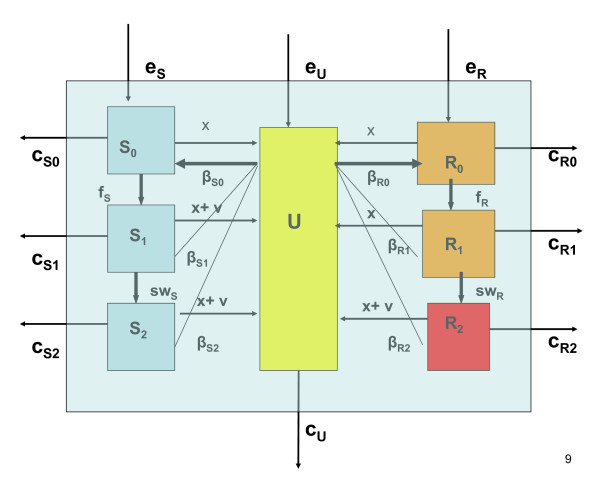
**Basic hospital model**. See the text and Table 1 for the definitions of the parameters and variables and a description of the model and the assumptions behind its construction.

The model we develop here is an extension of the model presented in a previous publication [5]. The formats of the deterministic and stochastic versions of this model are the same. We assume the hospital is a closed environment into which patients enter and leave. Within the hospital, patients are of different states with respect to colonization and treatment with one of two drugs for the target bacteria (e.g. *Staphylococcus *or *Enterococcus*). Patients are either uncolonized, ***U***, colonized with bacteria susceptible to both drugs, ***S***, or colonized with bacteria that are resistant to drug 1 but susceptible to drug 2, ***R***. In this basic model, we assume there is no resistance to the second drug (see the Discussion). On any given day, colonized patients carrying the S bacteria are of three states with respect to treatment, **S**_**0 **_untreated, ***S***_***1 ***_treated with drug 1, ***S***_***2 ***_treated with drug 2. Patients colonized with bacteria resistant to drug 1 are of the three corresponding states with respect to treatment with drugs 1 and 2: ***R***_***0***_, ***R***_***1 ***_and ***R***_***2***_. The letters, ***U, S, S***_***1***_, ..., are both the designations of these patient states and their numbers (proportions) in the hospital. See Figure 1 for a diagram of this model. The parameters and variables in this basic model are separately defined in Table 1.

**Table 1:** Parameters and their default values.

Symbol	Description	Value in Simulations
e_S_	Proportion colonized with sensitive bacteria among patients entering the hospital	0.40

e_R_	Proportion colonized with resistant bacteria among patients entering the hospital	0.0 - 0.2

*β*_S0_	Transmission rate of sensitive bacteria from an untreated patient	0.007

*β*_S1_	Transmission rate of sensitive bacteria from a patient treated with drug 1	0.007

*β*_S2_	Transmission rate of sensitive bacteria from a patient treated with drug 2	0.007

*β*_R0_	Transmission rate of resistant bacteria from an untreated patient	0.007

*β*_R1_	Transmission rate of resistant bacteria from a patient treated with drug 1	0.007

*β*_R2_	Transmission rate of resistant bacteria from a patient treated with drug 2	0.007

*f*_*S*_	Rate of initiating treatment with drug 1 for a patient in	varies

*f*_*R*_	Rate of initiating treatment with drug 1 for a patient in	varies

*sw*_*S*_	Rate of switching to drug 2 for a patient in	varies

*sw*_*R*_	Rate of switching to drug 2 for a patient in	varies

*x*	Rate of clearance for patients who are not treated or are treated with an ineffective drug	0.10

*v*	Additional rate of clearance for patients who are treated with an effective drug	0.50

*c*_*U*_	Rate of exiting hospital for patients in U.	0.20

*C*_*S*_	Rate of exiting hospital for patients in S.	0.10

*C*_*R*_	Rate of exiting hospital for patients in R.	0.10

All the rates are daily rates.

Untreated patients may begin treatment with antibiotic 1 at rates *f*_*S*_, and *f*_*R *_so that on any given day *f*_*S*_·*S*_*0 *_and *f*_*R*_·*R*_*0 *_untreated patients colonized with bacteria susceptible and resistant to antibiotic 1 begin treatment with that antibiotic and enter the S_1 _and R_1_, states respectively. In this basic model, we assume that the rate of treatment of colonized patients is independent of whether they are colonized with sensitive or resistant bacteria, and that antibiotic 2 is a second-line drug which is not used upon first treatment. Colonized patients treated with antibiotic 1, S_1 _and R_1 _however, can be switched to antibiotic 2, at rate sw_S _and sw_R _per day. Once a patient is treated with drug 2, treatment will go on until the patient clears the bacteria or until she/he is discharged from the hospital.

During their tenure in the hospital, uncolonized patients of the *U *state may become colonized at rates proportional to the numbers of colonized individuals of the different states. Once colonized, patients enter the untreated class of that state. For example, during the course of a day, (*β*_*S0*_·*S*_*0 *_+ *β*_*S1*_·*S*_*1 *_+ *β*_*S2 *_·*S*_*2*_)·*U *uncolonized patients will be colonized with bacteria that are susceptible to drug 1 and enter the untreated S_0 _state, and (*β*_*R0 *_·*R*_*0 *_+ *β*_*R1 *_·*R*_*1 *_+ *β*_*R2 *_·*R*_*2*_)·*U *will be colonized by bacteria resistant to drug 1 and enter the R_0 _state. The parameters, ***β***_***S0***_, ***β***_***S1***_, ***β***_***S2***_, ***β***_***R0***_, ***β***_***R1***_, and ***β***_**R2 **_are rates of infectious transmission [7] for patients of different states. In this basic model, we assume that patients colonized with susceptible or resistant bacteria, *S *or *R*, have to be cleared (enter the *U *state) before bacteria of the other kind can re-colonize them.

Patients may be cleared of the colonized bacteria, either spontaneously or through successful treatment, and enter the U state. The rates of spontaneous and antibiotic-mediated clearance are *x *and *v*, respectively. Patients colonized with bacteria resistant to antibiotic and treated with that antibiotic, *R*_*1*_, only clear those bacteria spontaneously.

New patients entering the hospital may enter into the states *U, S*_*0 *_or *R*_*0 *_at rates *e*_*U*_, *e*_*S *_and *e*_*R*_, respectively. We assume that individuals are not treated before they enter the hospital. Hospitalized patients may be discharged at rates that depend on their colonization status and treatment. The discharge rates are denoted by *c*_*X *_where *X *denotes the state is which the patient is just prior to discharge. The entrance and discharge rates are chosen so that, on average, the daily number of entering patients equals to the daily number of patients who are discharged.

### The deterministic model

The rates of change in the numbers of the different patient states are given by a set of coupled differential equations. For the numerical analysis of this deterministic model we use Berkeley Madonna™. Copies of the program used can be obtained from http://www.eclf.net/programs.

### The stochastic model

The main difference between the deterministic and stochastic version of this model are that the former considers the transitions at the population level. For example, in the deterministic model on any given day precisely *S*_*0 *_·*x *untreated patients colonized with susceptible bacteria enter the U state. The stochastic version of the model, on the other hand, keeps track of individual patients and in this example, the parameter *x *is the daily *transition probability *for an individual patient rather than a rate for the population at large Thus, a patient of the S_0 _state has a probability of *x *per day of being spontaneously cleared of the infection. The stochastic version of this model enables us to more precisely simulate processes that include several steps and to investigate the efficacy of different regimes of treatment and switching drugs, e.g. the effectiveness of switching a patient from drug 1 to drug 2 after a fixed number of days of treatment with drug 1 rather than switching at the same rate per day. The stochastic model can also be used to estimate quantities that are related to periods longer than one day. For example, the total number of days a patient was colonized with the resistant bacteria.

For the stochastic simulations we use a Monte Carlo protocol where each day we calculate the probability that each patient will move from her/is current state to each of the other states, or will stay at the same stage. A pseudo random number between 0 and 1 from a uniform distribution is generated and that patient's new state is determined by comparing this number with the transition probabilities. For example if the random number *y *is less or equal to *x*, then a colonized patient will enter the U state that day. If *y *>*x*, the patient will remain colonized. Once this has been done for all the patients, the simulation program determines and stores the total number of patients in each state on that day. This simulation program runs for a given number of days (currently 360 days). The outcome for one simulation is the average number of patients in each state over the last 100 days. This entire process is repeated many times (currently 200 times) and the final outcome is the average proportion (over the 200 simulations) of patients uncolonized, colonized with susceptible bacteria and colonized with resistant bacteria. It should be noted that because of their structural differences, and primarily the sequential order of events occurring on the same day (see Appendix), there are often modest quantitative differences between the outcomes predicted by the deterministic model and the average results of the stochastic simulations. This Monte Carlo simulation was programmed in Fortran and can be obtained from mhaber@sph.emory.edu.

## Results

### Deterministic simulations

**Figure 2:**
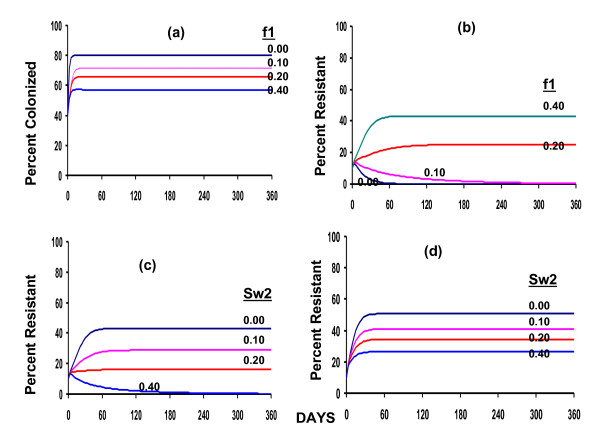
**Deterministic model, numerical solutions: Change in the number of colonized patients or the frequency of resistance for different treatment regimes**. (a) Change in the equilibrium frequency of patients colonized with susceptible bacteria for different frequencies of treatment with antibiotic 1. No resistance, and of the patients entering the hospital 60% are uncolonized and 40% are colonized with bacteria susceptible to antibiotic 1. The different curves correspond to different treatment rates which are in parentheses. Clearance rates with and without treatment are × = 0.10, v = 0.50, respectively, all colonized patients leave at the same rate, c_S _= c_R _= 0.10, and uncolonized patients leave the hospital at twice that rate, c_U _= 0.20. (b) Change in the number of patients colonized with bacteria resistant to antibiotic 1 with different frequencies of treatment with antibiotic 1. Parameters are the same as those in (a) but at the start of the simulation 10% of the patients in the hospital are colonized with resistant bacteria. (c) Change in the number of patients colonized with bacteria resistant to antibiotic 1 with different rates of switching to antibiotic 2. 40% of colonized patients are treated with antibiotic 1, fs = fr = 0.40 and antibiotic 1 and antibiotic 2 are equally effective on bacteria that are susceptible to their action. No input of resistant bacteria. Other parameters are the same as in previous figures. (d) Change in the number of patients colonized with bacteria resistant to antibiotic 1 with different rates of switching to antibiotic 2. 10% of the patients entering the hospital carry bacteria resistant to antibiotic 1, 40% are colonized with bacteria that are susceptible to antibiotic 1, and 50% are uncolonized. Other parameters are the same as in (c).

In an effort to orient the reader on the properties of the basic model and its general predictions, we use numerical solutions to the deterministic version of the model. The values of the parameters used for the numerical solutions were chosen to illustrate the properties of this model. Although we believe they are in a realistic range, they do not reflect estimates made in a specific hospital or treatment situation. In Figure 2(a), we illustrate what we would obtain with and without treatment in the absence of resistance. As long as there is an input of susceptible patients (*e*_*S *_> 0) there will be a stable equilibrium with U and S patients present. As noted in [5], the frequency of colonized patients will depend on the total density of patients in the hospital, the rates at which *S*_*0 *_and *S*_*1 *_patients are cleared and enter the U state or leave the hospital, their rates of infectious transmission, and rates of input of patients carrying susceptible bacteria.

Our model assumes that treatment augments the rate of clearance of bacteria from colonized patients. Hence, in the absence of resistance the equilibrium frequency of colonized patients will decline with the rate at which they are treated. If patients carrying resistant bacteria never enter the hospital (*e*_*R *_= 0) and resistant bacteria are not at a transmission and/or removal rate disadvantage (*β*_*S *_= *β*_*R *_and *c*_*S *_= c_*R *_), and in the absence of treatment, the frequency of hospitalized patients colonized with resistant bacteria will decline and the resistant bacteria will be eliminated (Figure 2b). If antibiotic 1 is used alone then there would be a threshold rate of use below which resistance will not ascend and above which the frequency of patients colonized with bacteria resistant to antibiotic 1 will increase. However even under these conditions, as long as patients with susceptible bacteria continue to enter the hospital there will be a polymorphism with uncolonized patients, patients colonized with bacteria resistant to drug 1, and patients colonized with bacteria susceptible to drug 1. The frequency of patients with bacteria resistant to drug 1 will be proportional to the rate at which this antibiotic is used.

In Figure 2c we consider the effects of switching to a second antibiotic for which there is no resistance on the frequency of patients colonized with bacteria resistant to antibiotic 1. The greater the rate of switching, the greater the rate and extent of reduction in the frequency of resistance to this first antibiotic. As can be seen in Figure 2d, this optimistic picture of being able to control the frequency and even eliminate resistance to drug 1 by using a second drug for which there is no resistance is thwarted if patients entering the hospital carry bacteria resistant to drug 1. Switching to the second drug is still effective in reducing the frequency of resistance to the first drug, but far less so than in the absence of input of patients colonized with bacteria resistant to the first drug.

### Stochastic simulations

The results of the deterministic simulation, like those of the model from whence it was derived [5], illustrates in a general way how switching hospitalized patients colonized with bacteria resistant to one antibiotic to a second one to which there is no resistance can reduce the overall frequency of resistance to the first drug. In the following, we use the stochastic version of this model to evaluate the relative efficacies of three protocols for switching patients to reducing the frequency of resistance to that first drug.

(1) Random switching: for each patient there is a constant daily probability of switching to a second drug. In essence, this is a stochastic version of the switching process considered in the deterministic model.

(2) Defined term switching: after a pre-determined number of days of treatment with the first drug, the patient is switched to the second drug.

(3) Directed switching: The patient remains on the first drug until testing indicates that the bacteria colonizing that individual is resistant to that drug, at which time that patient is switched to the second drug.

A list of all the parameters in our model and the values assigned to those parameters that remain fixed are presented in Table 1. Whenever possible the values of the parameters used in the stochastic simulation are the same as those in the deterministic simulations. For all situations we assume, probably realistically, that treatment is initiated before information is available about the resistance status of the colonizing bacteria. In other words, *f*_*S *_= *f*_*R*_.

### Random switching

**Figure 3:**
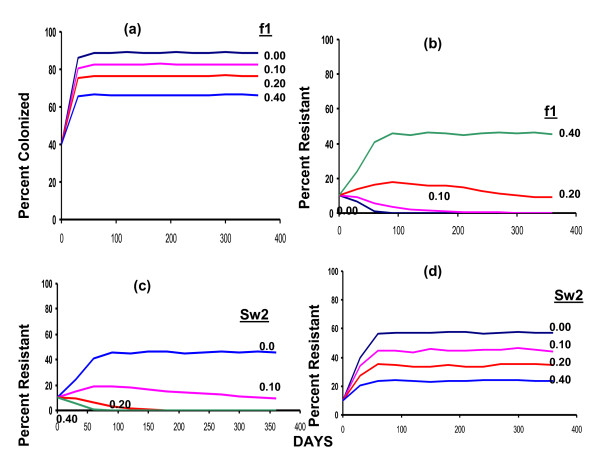
**Stochastic simulations**: (a) The effect of different rates of treatment with antibiotic 1 on the frequency of colonized patients in the absence of resistance. (b) The effects of different rates of treatment with antibiotic 1 on the frequency of patients colonized with bacteria resistant to this antibiotic without the use of antibiotic 2. Initially the frequency of patients colonized with bacteria resistant to antibiotic 1 is 10% and no patients enter the hospital carrying resistant bacteria. (c) The effect of different rates of random switching to a second drug on the frequency of resistance to the first antibiotic. Initially 10% of the patients are colonized with bacteria resistant to drug 1 and 40% of colonized patients are treated with drug 1, f = 0.40. No patients carrying bacteria resistant to drug 1 enter the hospital. (d) The effect of different rates of random switching to a second drug on the frequency of resistance to the first antibiotic. The parameters are the same as those in (c) save for the 10% of the patients entering the hospital carrying bacteria resistant to drug 1.

In Figure 3a we consider the relationship between the rate of use of drug 1 and the fraction of the population colonized by bacteria in the absence of resistance. The initial conditions and parameter values in this stochastic model are identical to those in the corresponding deterministic simulations presented in Figure 2. Except for small quantitative differences due to the reasons considered in the Appendix, the results of these stochastic simulations are the same as those of the corresponding deterministic simulations

In the absence of resistance, antibiotic treatment will reduce the frequency of colonized patients at a rate proportional to the rate of use (Figure 3a). If the hospital includes patients colonized with bacteria resistant to the antibiotic, the frequency of resistance can increase. As noted with the deterministic model (Figure 2b) the stochastic model predicts a threshold rate of antibiotic use below which resistance will not ascend and above which it will (Figure 3b). In the absence of input of patients colonized with bacteria resistant to antibiotic 1, random switching to a second antibiotic for which there is no resistance can reduce the frequency of patients colonized with bacteria resistant to the first antibiotic, and with a high enough rate of switching this procedure can eliminate resistant bacteria from the hospital (Figure 3c). With the input of patients carrying resistant bacteria however, switching to this second antibiotic can reduce the frequency of resistance but not eliminate resistance from the hospital (Figure 3d).

### The effects of different treatment regimes

For the comparison of the impact of different regimes for employing the second-line antibiotic on the total frequency of colonized patients and the frequency of patients colonized with bacteria resistant to the first drug, we consider these frequencies after one year from the initiation of treatment. As in Figures 3, within 100 days these frequencies remain relatively constant.

**Figure 4:**
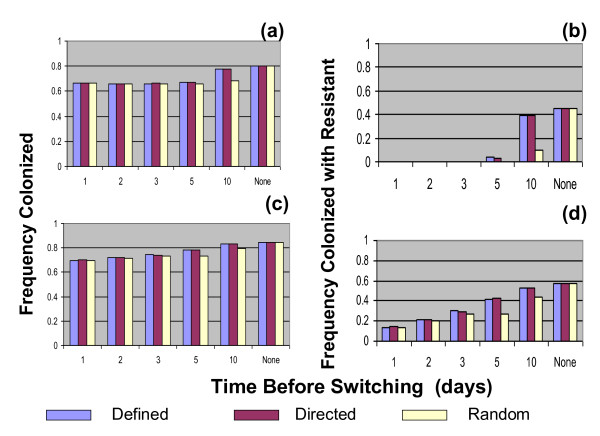
**Stochastic simulations**. Effects of different second line drug treatment regimes on the frequency of patients colonized with bacteria and the fraction colonize with bacteria that are resistant to the first line drug. Mean fractions for 200 runs after 1 year under that treatment regime. (a, b) No input of patients with resistant bacteria. (c,d) 10% of the patients entering the hospital carry bacteria resistant to the first-line antibiotic. Save for those related to switching, the values of the parameters of these simulations are the same as those in Figures 2 and 3. The standard errors were less than 1% of the mean for all sets of parameters.

In the absence of patients carrying resistant bacteria entering the hospital, the more rapidly patients are switched to the second drug, the fewer patients are colonized with bacteria, although there is little difference in this frequency if switching occurs within the first five days (Figure 4a). With respect to the fraction of patients colonized, save for late (10 days) switching, there is little difference in whether switching occurs at random, or according to other schemes. While switching to the second drug can clear the hospital of patients carrying resistant bacteria, it is less effective in doing so as the time before switching increases. Moreover, even with delayed switching, the random switching protocol is more effective in reducing the frequency of patients with bacteria resistant to the first drug than directed or defined period switching. The reason for this is that under random switching, there is a good chance that switching will actually occur earlier than the mean time to switching. For example, if the mean time to switching is 5 days then under random switching there is a chance of 59% that the patient will be switched during one of the first 4 days following onset of colonization and will clear infection soon after this. Under directed and defined switching, a patient colonized with bacteria resistant to drug 1 must wait 5 days until s/he is switched to drug 2, therefore this patient will, on average, stay longer in the 'resistant' state.

When patients with resistant bacteria enter the hospital, there is a modest increase in the frequency of colonized patients in the hospital relative to that without resistance entering. However, in this situation early switching to the second-line drug has a relatively greater effect on reducing the frequency of colonized patients (Figure 4c). While it's no longer possible to clear the hospital of patients with bacteria resistant to the first antibiotic, switching to an antibiotic for which there is no resistance reduces the frequency of patients with resistant bacteria. The more rapidly the switching occurs the lower the frequency of patients with resistant bacteria. Once again, random switching is more effective in this regard than directed or defined switching. The reason for this is the same as that described above.

## Discussion

Hospital-acquired infections are a major source of morbidity and mortality in the developed as well as the underdeveloped world, and a significant contributor to the ever-increasing costs of health care. Extrapolating from the data presented in the recent report of the Pennsylvania Health Care Cost Containment Council [8] to the United States at large, under the assumption of the same rates and costs (Pennsylvania is 4.2% of the USA population), we concluded that in 2005 there were more than 456,000 hospital-acquired infections in the USA. The frequencies of mortality of patients with and without these infections were 12.9% and 2.3%, respectively. In other words, in 2005 hospital-acquired infections contributed to approximately 48,000 excess deaths in the US. The term of hospitalization of patients with and without hospital-acquired infections were 20.6 and 4.5 days, respectively, for an excess cost of hospitalization due to hospital-acquired infections in the US at large being on the order of $70 billion in 2005. Needless to say, anything that can be done to reduce the incidence of hospital-acquired infections would be valuable from all perspectives. There is no reason or evidence to suggest that in general the hospital infection problem has abated in the past five years. There is also no reason to assume that unless a concerted effort is made to address this problem that the incidence of hospital-acquired infections and the human and economic costs they engender will wane in the future. There are, however, compelling reasons to believe that unless this effort is made, nosocomial infection will become increasingly difficult to deal with [9].

In this report we have focused primarily on the effects of switching to second-line antibiotics to reduce the incidence and term of hospital-acquired infections, especially infections cased by drug-resistant bacteria. However, before expanding on this pharmaceutical solution to a pharmaceutical problem, we believe it is essential ethically, as well as practically, to illustrate and emphasize the use of protocols that *reduce *rather than *increase *the use of drugs to deal with not only the resistance problem, but also to reduce incidence of hospital-acquired infections at large. As noted in our introduction, the antecedent of the model used here [5], as well as this model, predict that the same changes in hospital practices that reduce resistance will also reduce the incidence of hospital-acquired infections in general, a win-win situation. To illustrate this we use the deterministic version of the model in Figure 1 but allow for only three classes of patients, Uncolonized (U), Colonized with susceptible and untreated (S_0_) and colonized with susceptible and treated with antibiotic 1 (S_1_). In Table 2 we present the effects of changing specific parameters, one at a time, on the frequency of colonized patients at equilibrium.

**Table 2:** Equilibrium fraction of infected patients with different interventions

Intervention (changes in hospital protocol)	Percent of Infected Patients at Equilibrium
Standard Parameters*	52.8

Reduce transmission (β_S0_) by a factor of two	37.3

Reduce the term of stay of uncolonized patients (1/C_U_) by a factor of two	60.8

Reduce the term of stay of infected patients (1/C_S0_) by a factor of two	44.7

Reduce the input of colonized patients entering the hospital (e_S_) by a factor of two	43.3

Treat 50% of colonized patients with an antibiotic (f = 0.5) - no resistance	21.4

* Standard parameters: β_S0 _= 2 × 10^-3^, C_U _= 0.10, C_S0 _= 0.05, e_S _= 0.40, e_U _= 0.20

x = 0.10 (clearance rate in the absence of treatment - 10 days), v = 0.3333 (clearance rate with treatment - 3 days), β_S1 _= 2 × 10^-3 ^(transmission rate of treated patients with susceptible bacteria). Total and sustained number of patients N = 100. Note: A number of these parameters are different than those used in simulations presented in the body of this report.

Save for reducing the term of stay of uncolonized patients and thereby increasing the fraction of infected patients, all of these interventions reduce the absolute number of infected patients and all but one of these measures does not involve an increase in the use of antibiotics, just the opposite. The absolute magnitude of the effects of these different interventions on the frequency of infected patients depends, of course, on the values of the parameters. We chose these values for illustration rather than on the basis of estimates in real hospital and they are, we would like to think, an extreme on the negative side. At qualitative level, however, these effects of different interventions on the relative frequency of infected patients are, we believe, accurate for parameters in a realistic range.

Unfortunately, situations like that considered above, where all bacteria responsible for nosocomial infections are susceptible to the first-line antibiotics traditionally used for treatment are unlikely to be met in many hospitals. The reality that has to be dealt with is how to reduce the morbidity and mortality of individual patients who are likely to be infected with bacteria resistant to first-line antibiotics and the frequency of infected patients in the hospital at large. As illustrated in Table 2, even with high frequencies of patients infected with antibiotic resistant bacteria, goals can be achieved without the use of second-line antibiotics by: (i) improving infection control measures, like promoting hand washing and the more effective decontamination of equipment and surfaces and medical devices like catheters and respirators, (ii) quarantining or otherwise preventing patients already colonized with these bacteria from entering intensive care wards or areas of hospitals where patients are particularly prone to bacterial infections and are most likely to manifest serious symptoms, and (iii) increasing the rate of discharge of colonized and infected patients.

How many of these interventions can be implemented and the extent to which they can be implemented remains to be seen. It is, however, certain that substantial improvements can be made. This is very clear from the Pennsylvania experience [8]: the incidence of infections varies considerably among hospitals. To be sure, some this variation can be attributed to the variation in the patient population, but not all of it.

With respect to the contribution of second-line drugs to reducing the incidence of infections with bacteria resistant to the first-line drugs, the results of the present investigation with a stochastic simulation model are, as would be anticipated, qualitatively consistent with those anticipated from the analysis of the deterministic model [5]. As long as the bacteria remain susceptible to these second-line drugs, switching to those drugs can reduce the frequency of colonized patients at large and the frequency of patients carrying bacteria resistant to these agents. In the absence of patients carrying resistant bacteria entering the hospital, switching to the second-line drug can not only reduce the frequency of patients with bacteria resistant to the first-line drug but actually eliminate those bacteria. If patients carrying resistant bacteria enter the hospital, then the elimination of resistance cannot be achieved and it will not even be possible to reduce the incidence to that which is coming in. However, switching to the second-line drug can still reduce the frequency of patients carrying bacteria resistant to the first-line drug. Whether resistance enters or not, the earlier the switching is done the lower the frequency of patients carrying resistant bacteria.

Our results suggest no difference in the contribution of switching to second-line drugs to reducing the frequency of resistance to the first-line drug for two of the three switching regimes considered: defined term and directed switching. In the former regime switching occurs after a specified number of days while under the latter regime patients are switched after being identified as carrying bacteria resistant to the first-line drug. Overall, the third regime considered here, namely stochastic (random) switching, appeared to be the most effective of the three. In this regime, there is a constant daily probability of switching. The reason why random switching is more effective than defined-term switching can be seen in Figure 4b and 4d. The efficacy of switching declines with the time before switching occurs, with early switching contributing more than later switching. With stochastic switching, although the average time to switching may equal to a given number of days, actual switching is more likely to occur earlier than the average time because the distribution of time to switching is very skewed. Since early switching is more effective than late switching, stochastic switching is more effective in reducing resistance than defined term switching when the average number of days to switching are identical in both regimes.

In our model, directed switching does not turn out to do better than the other two mechanisms because we did not take costs into account. In general second-line drugs are more expensive than the first-line drugs. Under random and defined switching, many patients who are colonized by bacteria sensitive to the first drug would be unnecessarily switched to the second drug. On the other hand, under directed switching only patients colonized by the resistant bacteria are switched, i.e., the second drug is used only when it is needed. Thus, using a second drug when it is not needed will usually increase the cost of treatment and the likelihood of resistance to that drug. Although much of treatment failure may be due to factors other than inherited antibiotic resistance [10], some failure is indeed due to bacteria being inherently resistant to the treating drug, which should be the primary reason for switching. A thorough analysis of all the costs and benefits is needed in order to decide which of the switching modes is the most cost-effective.

In this model, we have assumed there is no resistance to the second line drug. Clearly if there is resistance to this drug, its efficacy in controlling the incidence of infections and reducing the incidence of treatment failure due to resistance to first-line drug would be compromised. As the frequency of resistance to this second line drug increases, as it almost certainly will with increasing use of that antibiotic, that proposed switching strategies could lose their effectiveness. Saying this in another way, the use of a second line drug to deal with resistance to first line antibiotics is only a transient solution to this problem. We could expand our model to include third and fourth-line drugs, as well as multiple switching strategies, but we believe that our conclusions would not be very different. We certainly do not endorse practices that endanger the health and well-being of individual patients by withholding antibiotics because of concerns about resistance. On the other hand, we believe that infection control preventing colonization with pathogenic bacteria remains the most long-term effective measure to reduce both the incidence of hospital-acquired infections in general and the spread of resistance to the antibiotics used for treatment and prophylaxis.

How good are the theoretical predictions about the consequences of different interventions on the incidence of hospital-acquired bacterial infections and resistance to first-line antibiotics? Although the stochastic version of this model is more realistic than the deterministic, both models can be described as simplistic caricatures of the complexities of hospitals. Although the parameter values used for the numerical analyses of the properties of these models may be in a 'realistic range', they are not estimates obtained from real hospitals and even if they were, they would only represent a small subset of the vast numbers of parameters that govern the dynamics of infectious disease transmission and treatment in hospital settings. While we appreciate and accept the limitations of these models and our analysis of their properties, we believe that at more qualitative rather than quantitative level the results and interpretations we make in this report are correct.

In summary, the number of hospital-acquired infections, the excess mortality and costs of these infections, as well as the spread of antibiotic resistance in hospitals can be significantly reduced by: (i) controlling the entry of patients colonized with bacteria (and other microparasites) that can be transmitted within a hospital into the main wards and particularly in the intensive care units, (ii) more intense and strictly enforced measures to reduce transmission of microbes between patients, from health care workers and from catheters and mechanical devices, (iii) reducing the term of hospitalization of infected patients, and (iv) switching to first and second-line drugs for which there is little or no resistance. The strategies for the application of each of these interventions can be, and we of course believe should be, examined with realistic mathematical and computer simulation models analyzed using parameters estimated in hospitals. As well illustrated by the studies [11,12], what may seem like a good strategy from purely intuitive arguments, like cycling antibiotics, may well not be the optimal.

## Competing interests

The authors declare that they have no competing interests.

## Authors' contributions

BRL and MH wrote the manuscript and conducted the deterministic and stochastic simulations, respectively. PK helped with important comments related to the administration of antibiotic-resistant drugs. All authors read and approved the final manuscript.

## Appendix: Explanation for the Differences between Deterministic and Stochastic Simulation Results

The difference between simulation results obtained from the two methods results from the way they deal with multiple events occurring in the same time interval, which is one day in our case. The transition rates used in deterministic model correspond to probabilities that ignore the possibility of two or more events occurring during the same time interval. Hence these rates correspond to *unconditional probabilities*. In the stochastic model the probability of an event depends on other events that have happened during the same time interval, hence this model deals with *conditional probabilities*.

For example, consider an uncolonized patient. There are two things that may happen to this patient on a given day. He may exit the hospital (denote this event by *E)*, and he may get colonized (event *C*). Suppose that the probabilities of *E *and *C *are 0.2 and 0.6, respectively. These probabilities determine the transition rates in the deterministic model. The stochastic model, on the other hand, argues that if a patient exits the hospital he cannot be become colonized (and even if he becomes colonized he cannot infect other patients). Thus, the stochastic model considers 0.6 as the *conditional *probability of the patient becoming colonized *if he stays in the hospital*, i.e., if the event *E *has not occurred on the same day.

We denote by *P*(*C*) the unconditional probability of the event *C*, and by *P*(*C *| *E*) the conditional probability of the event *C *when it is known that the event *E *has occurred. We also denote by *Ē *the event '*E *has not occurred', and by *P*(*C *| *Ē*) the probability that *C *has occurred when it is known that *E *has not occurred. Then a well-know probability theorem relates the conditional and unconditional probabilities as follows:

*P*(*C*) = *P*(*C *| *E*) × *P*(*E*) + *P*(*C *| *Ē*) × *P*(*Ē*). In out example, *P*(*C *| *E*) = 0, *P*(*E*) = 0.2, *P*(*C *| *Ē*) = 0.6, *P*(*Ē*) = 0.8. Hence the unconditional probability in the stochastic model is 0 × 0.2 × 0.6 × 0.8 = 0.48, rather than the 0.6 used by the deterministic model.

## Pre-publication history

The pre-publication history for this paper can be accessed here:

http://www.biomedcentral.com/1471-2334/10/254/prepub

## References

[B1] Levy SB Marshall B Antibacterial resistance worldwide: causes, challenges and responses Nat Med 2004 10 S122 S129 10.1038/nm1145 15577930

[B2] Diekema DJ BootsMiller BJ Vaughn TE Woolson RF Yankey JW Ernst EJ Flach SD Ward MM Franciscus CL Pfaller MA Doebbeling BN Antimicrobial resistance trends and outbreak frequency in United States hospitals Clin Infect Dis 2004 38 78 85 10.1086/380457 14679451

[B3] Rubin RJ Harrington CA Poon A Dietrich K Greene JA Moiduddin A The economic impact of Staphylococcus aureus infection in New York City hospitals Emerg Infect Dis 1999 5 9 17 10.3201/eid0501.990102 10081667 PMC2627695

[B4] EARSS Annual Reports 2004 http://www.rivm.nl/earss/result/Monitoring_reports/#tcm:61-25397

[B5] Lipsitch M Bergstrom CT Levin BR The epidemiology of antibiotic resistance in hospitals: paradoxes and prescriptions Proc Natl Acad Sci USA 2000 97 1938 1943 10.1073/pnas.97.4.1938 10677558 PMC26540

[B6] Karchmer TB Durbin LJ Simonton BM Farr MB Cost-effectiveness of active surveillance cultures and contact/droplet precautions for control of methicillin-resistant Staphylococcus aureus J Hosp Infect 2002 51 126 132 10.1053/jhin.2002.1200 12090800

[B7] Anderson RM May RM Infectious Diseases of Humans: Dynamics and Control 1991 Oxford: Oxford University Press

[B8] PHC4. Hospital Acquired Infections in Pennsylvania 2006 http://www.phc4.org/reports/hai/05/docs/hai2005report.pdf

[B9] Torres C Up against the wall Nat Med 2010 16 628 631 10.1038/nm0610-628 20526310

[B10] Levin BR Rozen DE Non-inherited antibiotic resistance Nat Rev Microbiol 2004 4 556 562 10.1038/nrmicro1445 16778840

[B11] Bergstrom CT Lo M Lipsitch M Ecological theory suggests that antimicrobial cycling will not reduce antimicrobial resistance in hospitals Proc Natl Acad Sci USA 2004 101 13285 13290 10.1073/pnas.0402298101 15308772 PMC516561

[B12] Bonhoeffer S Lipsitch M Levin B Evaluating treatment protocols to prevent antibiotic resistance Proc Natl Acad Sci USA 1997 94 12106 12111 10.1073/pnas.94.22.12106 9342370 PMC23718

